# Exploring Aurone Derivatives as Potential Human Pancreatic Lipase Inhibitors through Molecular Docking and Molecular Dynamics Simulations

**DOI:** 10.3390/molecules25204657

**Published:** 2020-10-13

**Authors:** Phuong Thuy Viet Nguyen, Han Ai Huynh, Dat Van Truong, Thanh-Dao Tran, Cam-Van Thi Vo

**Affiliations:** Faculty of Pharmacy, University of Medicine and Pharmacy at Ho Chi Minh City, Ho Chi Minh City 700000, Vietnam; hanhuynh1995@gmail.com (H.A.H.); dattv@ump.edu.vn (D.V.T.); daott@ump.edu.vn (T.-D.T.)

**Keywords:** aurone, human pancreatic lipase, human pancreatic lipase inhibitors, molecular docking, molecular dynamics simulations

## Abstract

Inhibition of human pancreatic lipase, a crucial enzyme in dietary fat digestion and absorption, is a potent therapeutic approach for obesity treatment. In this study, human pancreatic lipase inhibitory activity of aurone derivatives was explored by molecular modeling approaches. The target protein was human pancreatic lipase (PDB ID: 1LPB). The 3D structures of 82 published bioactive aurone derivatives were docked successfully into the protein catalytic active site, using AutoDock Vina 1.5.7.rc1. Of them, 62 compounds interacted with the key residues of catalytic trial Ser152-Asp176-His263. The top hit compound (**A14**), with a docking score of −10.6 kcal⋅mol^−1^, was subsequently submitted to molecular dynamics simulations, using GROMACS 2018.01. Molecular dynamics simulation results showed that **A14** formed a stable complex with 1LPB protein via hydrogen bonds with important residues in regulating enzyme activity (Ser152 and Phe77). Compound **A14** showed high potency for further studies, such as the synthesis, in vitro and in vivo tests for pancreatic lipase inhibitory activity.

## 1. Introduction

Industrialization, urbanization, and modernization have shifted our lifestyle into an inactive one. Convenient processed food with a high degree of fat and free energy sugar exists more often in our daily diets. With these changes, the global obese and overweight population have almost tripled since 1975 [1]. Obesity, a complicated and multifactorial disease, mostly arises from the imbalance between energy intake and energy consumption in the body. Obesity elevates the risk of other serious diseases, listing as diabetes [2,3], cardiovascular disorders [4,5], and certain cancers [6]. Current FDA-approved drugs for obesity treatment are orlistat, phentermine/topiramate, naltrexone/bupropion, and liraglutide [7,8]. Of them, orlistat is the only over-the-counter medication with the mechanism of inhibiting human pancreatic lipase (HPL)—enzyme hydrolyzing triglycerides in the digestion and absorption of dietary fat [9]. The limitations of orlistat are its adverse and inconvenient side effects, such as flatulence, steatorrhea, nephrotoxicity, kidney stones, and pancreatitis [7]. Since orlistat, there have been no new drugs getting approval with this mechanism. The inhibition of HPL to limit dietary fat digestion and absorption has thereby received much interest in anti-obesity drug discovery.

**Figure 1:**
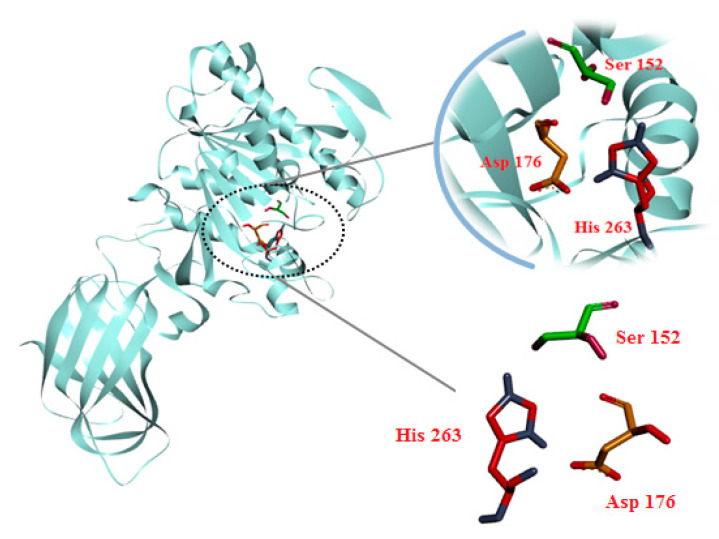
Three-dimensional structure of human pancreatic lipase and catalytic triad of Ser152-Asp176-His263 (PDB ID: 1LPB).

The crystal structure of HPL (PDB ID: 1LPB) was determined by X-ray diffraction. HPL is a glycoprotein consisting of the N region (residues from 1 to 335) and the C region (residues from 336 to 449), associated with a colipase [10,11]. In the N region, the active site consists of residues from 247 to 258 with the catalytic trial Ser152-Asp176-His263 (Figure 1). The lid domain is a surface loop made by a disulfide bridge between residues Cys237 and Cys261. The C region has a double-ring structure and provides the main binding surface for colipase. There are also two hair-loop coils from amino acids 76 to 85 and β9-loop from amino acids 204 to 224 [10]. HPL structure has two states: closed state and open state. In the open state, the lid domain binds via van der *Waals* interaction with β5-loop and β9-loop coils opening the catalytic site of the enzyme [12,13]. A C11 alkyl phosphonate (methoxy undecyl phosphonic acid (MUP)) was co-crystallized in the active site of HPL as a lipase inhibitor [12]. FDA-approved orlistat was also known as an irreversible inhibitor of pancreatic and gastric lipase by forming a covalent bond with the lipase active site in the lumen of the digestive tract [14,15].

Considerable effort in recent years has been devoted to the discovery of new pancreatic lipase inhibitors. Natural plant-derived compounds (alkaloids, saponins, carotenoids, glycosides, polyphenols, polysaccharides, and terpenoids) and microorganism-derived compounds (lipstatin, valilactone, and panclicins) have been isolated and reported to inhibit in vitro and in vivo pancreatic lipase [16,17,18]. Synthetic compounds with diverse structures have been prepared and screened for pancreatic lipase inhibitory activity [19,20,21,22]. Along with the conventional approaches, in silico models such as 3D QSAR, 2D pharmacophore, molecular docking, and molecular dynamics simulations are utilized to identify potential bioactive compounds for obesity treatment [23,24,25,26]. Flavonoids with subclasses of flavone, flavonone, and chalcone were identified as potential candidates. IC_50_ values of some structures were determined, notably licochalcone A (IC_50_ 35.00 µg/mL) [27], galangin (IC_50_ 48.20 mg/mL) [28], hesperidin (IC_50_ 32.00 µg/mL) [29], etc. Furthermore, 36 compounds with 1*H*-inden-1,3,5,6-tetrol structure have been designed, combined with molecular docking and molecular dynamics simulations, to gain three compounds binding well to the target. These compounds were considered as potential pancreatic lipase inhibitors [25]. In a different study, four compounds, namely sanggenon C, sanggenon D, kuwanin C, and kuwanon G, extracted from *Cortex Mori Radicis*, showed good HPL inhibition results with IC_50_ range of 0.77 to 20.56 µM and Ki less than 5.0 µM. In particular, sanggenon D had the strongest inhibitory activity with IC_50_ of 0.77 µM and Ki of 0.43 µM. Molecular docking was also performed between sanggenon D and HPL, which resulted in good binding to the active site through hydrogen interactions with Ser152 and hydrophobic interactions with Phe77 [30].

**Figure 2:**
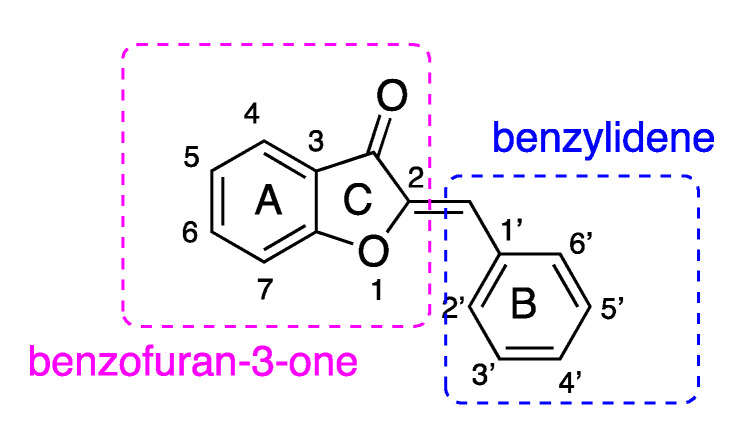
General structure of aurones.

Aurones (2-benzylidene-1-benzofuran-3(2*H*)-ones) (Figure 2) belong to a minor subclass of natural flavonoids [31]. Aurones have two isomers: (*Z*) and (*E*). Of them, (*Z*) isomers are experimentally more favored [32,33]. Aurones present widely in flowers and fruits. They play a crucial role in the pigmentation, typically bright yellow. They are also considered phytoalexins produced by plants in response to pathogen attack [33]. Aurone derivatives have been reported to possess different bio-activities [34], such as anti-inflammatory [35], antibacterial [36], anti-malarial [37,38], anti-hepatitis [39,40], antioxidant [41,42,43,44], anticancer [45,46,47,48,49], and others. However, their ability to inhibit HPL has not been studied.

In this article, we describe the exploration of aurone ability to inhibit human pancreatic lipase using molecular modeling approaches. Molecular docking on the HPL active site of the general structure of aurones, orlistat, and co-crystallized ligand was carried out as a preliminary study to determine their similarity in structures. The structures of 82 bioactive aurone derivatives were then docked into the same domain. The aurone structure with the highest docking score and proper interaction with the catalytic triad was submitted to the molecular dynamics (MD) simulations. The MD simulations of the best binding derivative and pancreatic lipase complex were performed to elucidate the dynamics behavior of the interactions between protein and ligand.

## 2. Results and Discussion

### 2.1. Molecular Docking

#### 2.1.1. Validation of Docking Protocol

The docking protocol was validated by redocking MUP, the co-crystallized inhibitor, into the active site of HPL. Initially, ligand MUP was retracted from the active site. It was subsequently redocked into the same domain. All conformations of MUP were located in the active site; the best conformation formed the interactions with HPL through the hydrogen bonds with Phe77, Ser152, Leu153, and His263. The resemblance of the MUP lowest binding energy pose and the experimental structure determined by X-ray crystallography proved that it was successfully mimicking the native pose. Root mean square deviation (RMSD) between the docked structure and the initial structure, using only movable heavy atoms (i.e., only ligand atoms, not hydrogen), was 0.86 Å (less than 2.00 Å). Thus, docking and redocking results of MUP signified that AutoDock Vina software is reliable [50]. The protocol, therefore, can be used for docking other compounds.

#### 2.1.2. Comparison of the General Structure of Aurones, Orlistat, and MUP

The general structure of aurones (**A0**) (Figure 2) and orlistat were docked into the same domain for comparative purpose. MUP, orlistat, and **A0** had the docking scores of −4.3, −6.7, and −8.8 kcal⋅mol^−1^, respectively. Of three compounds, **A0** docked best (Table 1).

**Figure 3:**
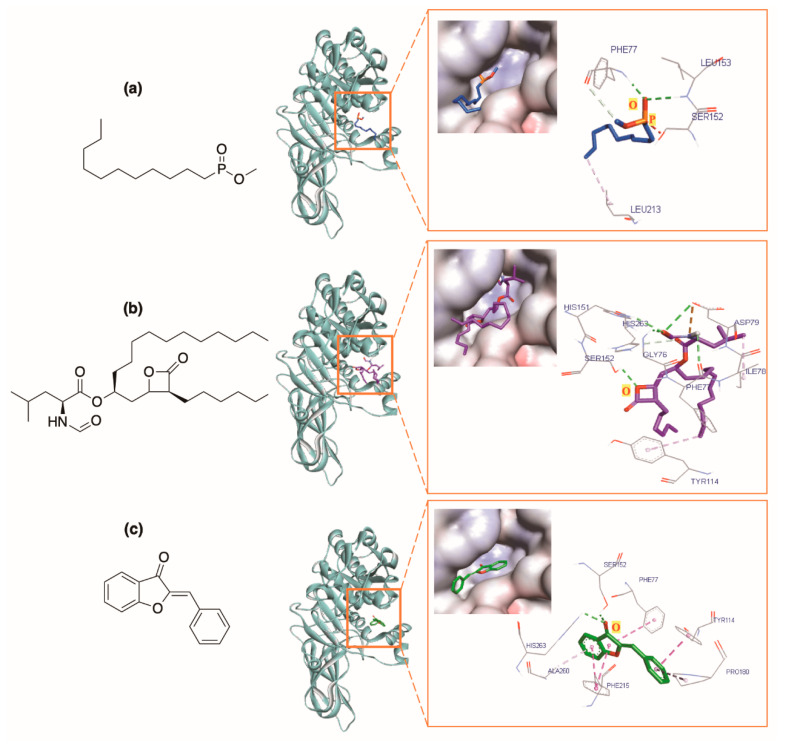
Docking results with protein (PDB ID: 1LPB) of (**a**) co-crystallized ligand MUP, (**b**) orlistat, and (**c**) general structure of aurones (**A0**). Lipase in blue, lipase active site residues in stick model.

MUP bound to the active site with four hydrogen bonds formed by the phosphonic group of MUP with Ser152, Phe77, Leu153, and His263 of HPL. Orlistat showed strong binding to HPL with five hydrogen bonds and different hydrophobic interactions. The lactone ring and the amino acid side chain of orlistat played the key role in forming these five hydrogen bonds with residues Ser152, Phe77, His151, Asp79, and Gly76. In the case of **A0**, the benzofuranone ring (rings A and C) were crucial in forming two hydrogen bonds with residues Ser152 and His263. The benzofuranone ring of aurone also made the π-π interactions with the aromatic rings of residues Phe77 and Phe215. The benzylidene ring (ring B) interacted with the aromatic ring of Tyr114 through the π-π interaction and hydrophobic interaction with Pro180 (Figure 3). The interaction profiles of **A0**, orlistat, and MUP with HPL indicated their structural similarity in the enzyme active site. They all interacted with the amino acids of the catalytic trial (Ser152-Asp176-His263) and Phe77, an important amino acid for lipase activity [10,51,52]. These structural similarities hint the ability of aurones to interact with the active site of HPL.

#### 2.1.3. Molecular Docking with Aurone Derivatives

The 3D structures of 82 bioactive aurone derivatives collected from previous publications were docked into the active site of HPL [34,35,36,37,38,39,40,41,42,43,44,45,46,47,48,49]. Conformations of these compounds were ranked according to their binding affinities on HPL. All investigated aurone derivatives successfully bound to HPL with good docking scores, ranging from −10.6 to −7.4 kcal⋅mol^−1^ (Table S1). Of them, 45 compounds (55%) had the scores less or equal to −9.0 kcal⋅mol^−1^, and 37 compounds (45%) had the docking scores greater than −9.0 kcal⋅mol^−1^.

**Figure 4:**
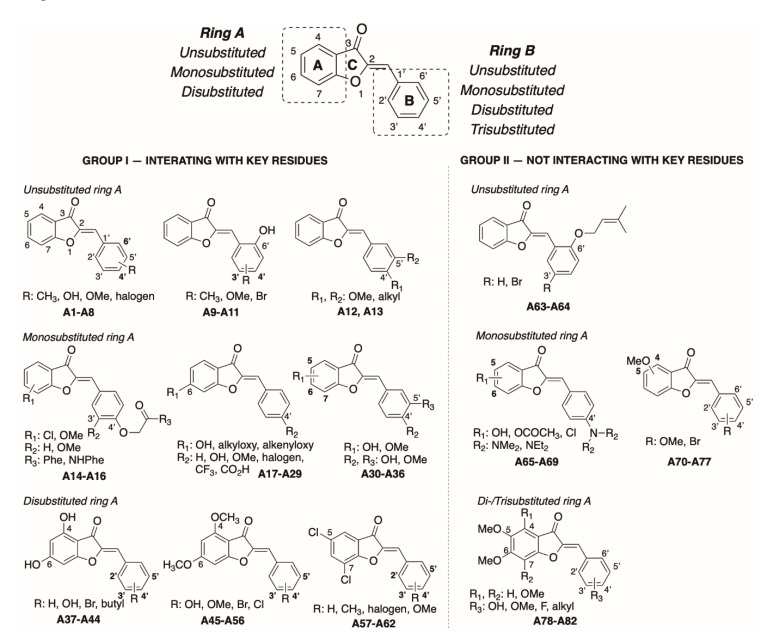
Classification of 82 aurone derivatives.

To better understand the binding mode of different aurone derivatives, we analyzed the interaction profiles of investigated compounds in addition to docking scores. Of 82 docked compounds, 62 compounds formed hydrogen bonds with the key residues of the catalyst trial Ser152-Asp176-His263 and Phe77 (Group I); 20 compounds did not have significant interactions with the key residues (Group II). For the ease of analyzing interaction profiles, Group I and Group II were further classified according to the substituent patterns of ring A (unsubstituted, monosubstituted, and disubstituted ring A) and ring B (unsubstituted, monosubstituted, and di/trisubstituted ring B) (Figure 4).

##### Group I—Interacting with Key Residues

*Unsubstituted ring A:* Aurones of this group differ in substituent pattern of ring B: (1) monosubstitution with halogen, hydroxy, methoxy groups at *ortho*/*para* positions, and (2) disubstitution with hydroxy, methoxy, alkyl groups. Docking scores were in the range of −8.8 to −10.5 kcal⋅mol^−1^ (Table 2).

**Figure 5:**
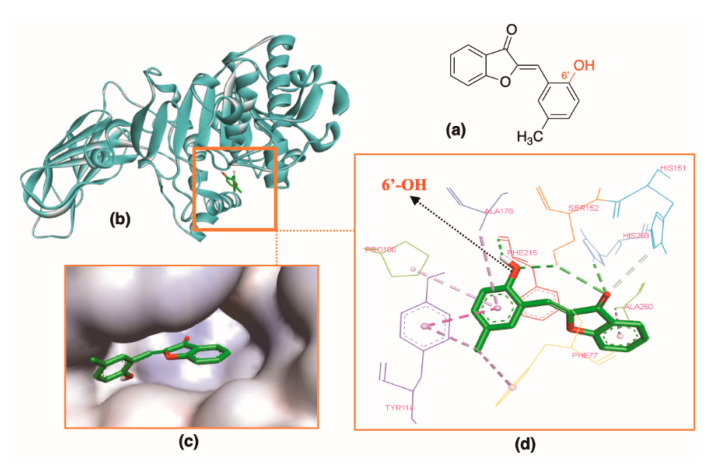
Docking result of **A11** with protein (PDB ID: 1LPB): (**a**) 2D structure of **A11**. (**b**) **A11** inside the active site in the ribbon style. (**c**) **A11** in the active site surface. (**d**) Interactions of **A11** and enzyme residues with hydrogen bonds in green and hydrophobic interactions in purple.

Aurones with monosubstitution on ring B (**A1**–**A8**) were fully located in the pocket. They all have a small substituent on ring B, including OH, CH_3_, OMe, and halogen (F, Cl, Br), and were not much different from the general structure of aurones (**A0**). They interacted with HPL in the same fashion of **A0** in which most interactions occurred at the benzofuranone ring (rings A and C) through hydrogen bonds and hydrophobic interactions with the residues Phe77, Ser152, His263, and Tyr114. Aurones with disubstitution on ring B (**A9**–**13**) docked well into the HPL active site, having the docking scores of −8.6 to −10.5 kcal⋅mol^−1^. Particularly, compounds with -OH group at 6′ position (**A9**–**A11**) interacted with HPL with the docking scores of −9.5 to −10.5 kcal⋅mol^−1^. Two hydrogen bonds were formed by the 3-ceton group of ring C and residues Ser152 and His263. The polar 6′-OH stayed in a favorable position and provided an additional hydrogen bond with Ser152. When we replaced 6′-OH (**A11**) with 6′-OMe (**A6**), the docking score declined. Of them, **A11** had the highest docking score (−10.5 kcal⋅mol^−1^) (Figure 5).

In the case that ring B consists of two adjacent substituents (**A12** and **A13**), a part of the structure was outside the catalytic cavity, and fewer hydrogen bonds with the key residues were formed compared to **A0**.

**Figure 6:**
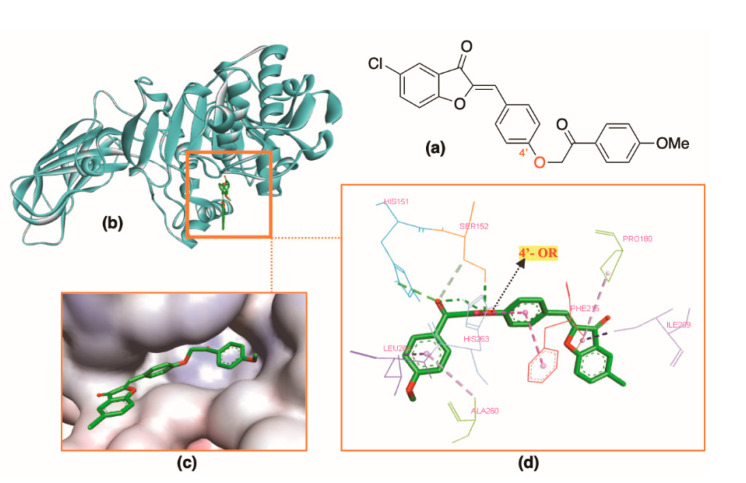
Docking result of **A14** with protein (PDB ID: 1LPB): (**a**) 2D structure of **A14**. (**b**) **A14** inside the active site in the ribbon style. (**c**) **A14** in the active site. (**d**) Interactions of **A14** and enzyme residues with hydrogen bonds in green and hydrophobic interactions in purple.

*Monosubstituted ring A:* Aurones of this group interacted with the active site in different manners. Aurones **A14**–**A16** with ring B oxy-tethering to a functionalized aromatic ring interacted with the active site not at ring A, as in the case of general aurone structure (**A0**). The bulky substituent at 4′ position lengthens the compound size, pushing the benzofuranone ring to slide out of the catalytic cavity, away from the main residues and into the hydrophobic region. Hydrogen bonds were alternatively created by ether oxygen atom with residues Ser152 and His263. This subgroup interestingly possessed high docking scores (−9.9 to −10.6 kcal⋅mol^−1^) (Table 2). Of which, structure **A14** with *Z*-isomer conformation docked best into the HPL binding site (−10.6 kcal⋅mol^−1^) and interacted with the key residues of the catalytic trial (Ser152 and His263) (Figure 6).

Aurones **A17**–**A29** with one substituent at the 6 position on ring A and one substituent at the 4′ position on ring B. Docking scores were −8.3 to −10.2 kcal⋅mol^−1^. When a substituent on ring A was small, such as OH and OMe **(A17**–**A26)**, aurones were located fully in the active site of HPL. When substituent on ring A is larger such as ethoxy (**A27**), dimethylallyloxy (**A28**), or prenyloxy (**A29**), ring A slid out of the catalytic cavity, away from the key residues and in contact with the hydrophobic region.

Aurones **A30**–**A36** possessed two adjacent substituents (OH and OMe). Similar to aurones with unsubstituted ring A and two adjacently disubstituted ring B, a part of aurones was outside the catalytic cavity, and fewer hydrogen bonds with the key residues were observed. The docking scores of these compounds were −8.4 to −9.1 kcal⋅mol^−1^.

**Figure 7:**
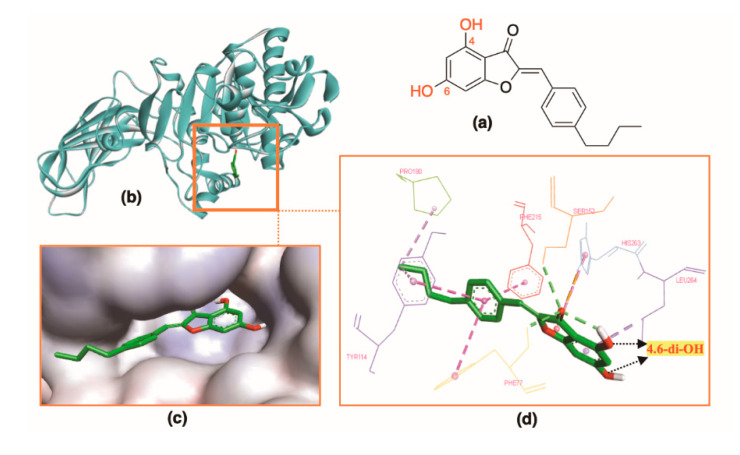
Docking result of **A42** with protein (PDB ID: 1LPB): (**a**) 2D structure of **A42**. (**b**) **A42** inside the active site in the ribbon style. (**c**) **A42** in the active site surface. (**d**) Interactions of **A42** and enzyme residues with hydrogen bonds in green and hydrophobic interactions in purple.

*Disubstituted ring A:* 4,6-Disubstituted benzofuranone aurones (OH and OMe) had a good shape and fit well in the active site. Compounds with 4,6-dihydroxy substituents (**A37**–**A44**) formed two hydrogen bonds with Phe77 and Ser152 by the C=O group of ring C and one hydrophobic interaction with His263. An intramolecular hydrogen bond was formed between 4-OH and 3-C=O. This interaction might help 4,6-dihydroxy aurones in better shape for binding into HPL. When 4,6-dihydroxy groups (**A37**) changed to 4,6-dimethoxy groups (**A45**), the docking scores decreased. In this group, compound **A42** (−10.5 kcal⋅mol^−1^) was the structure binding best to the HPL (Figure 7).

Aurones with 5,7-dichlorobenzofuranone (**A57**–**A62**) interacted with Ser152 and His263 by hydrogen bonds and with Leu264, Arg256, and Ala259 through the hydrophobic interactions. Docking scores varied from −8.2 to −10.1 kcal⋅mol^−1^.

##### Group II—Not Interacting with Key Residues

**Figure 8:**
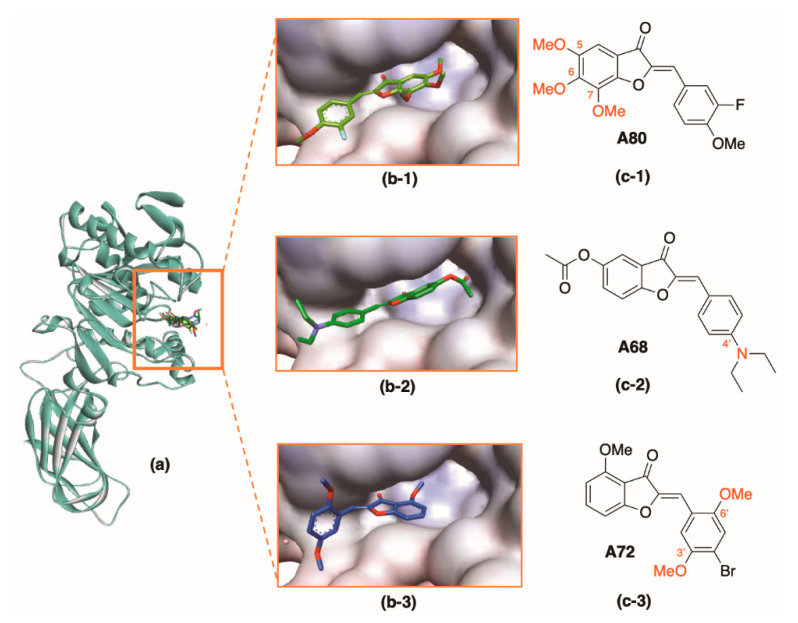
Some compounds of Group II in lipase (PDB ID: 1LPB) active site: (**a**) **A80**, **A68**, and **A72** inside the active site in the ribbon style. (**b**) **A80**, **A68**, and **A72** inside the active site. (**c**) 2D structures of **A80**, **A68**, and **A72**.

Docking scores of the remaining 20 aurone derivatives were in a medium-to-good range (−7.4 to −10.1 kcal⋅mol^−1^) (Table 2). These compounds, however, did not interact with the key residues of the active site (Figure 8). Compounds of Group II had many adjacent methoxy substituents or branched substituents in common. These adjacent groups made the compounds bulkier; therefore, they made it difficult to penetrate the catalytic pocket. There were mostly hydrophobic interactions formed with the key residues regulating enzyme activity (Ser152, His163, Asp176, and Phe77).

**Figure 9:**
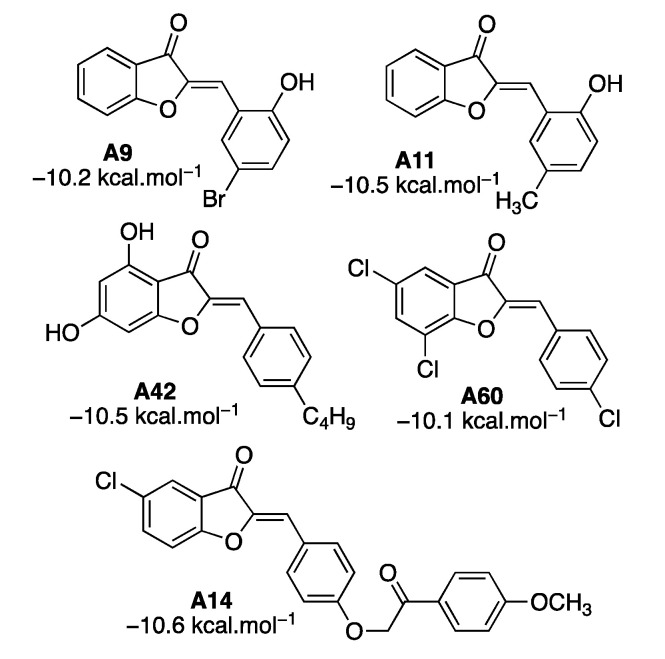
Compounds with the top docking scores and interacting with the key residues of human pancreatic lipase (HPL).

Overall, compounds (**A9**, **A11**, **A14**, **A42**, and **A60**) possess good docking scores and interact with the key residues of HPL (Figure 9). In terms of structures, they all have in common the oxygen-related substituents (OH, OMe, and oxy-tether to the aromatic ring). The presence of the -OH group at *ortho* position on ring B (**A11** and **A9**) much improves the binding affinity by forming additional hydrogen bonds with Ser152. Aurone **A42** with 4,6-dihydroxy groups on ring A formed an intramolecular hydrogen bond enhancing its binding. **A14** is an exceptional case. The bulky oxy-tether aromatic ring pushed the benzofuranone (rings A and C) out of the catalytic cavity, leaving ring B to interact with the catalytic trial Ser152-Asp176-His263 through hydrogen bonds. Interestingly, compound **A14** still had the top docking score (−10.6 kcal⋅mol^−1^). Moreover, **A14** was reported as a leukemia-cell-resistance agent [49]. Therefore, **A14** was selected for further study, to investigate the dynamics of the protein and ligand complex.

### 2.2. Molecular Dynamics Simulations

Protein–ligand docking provides only static interactions of protein and ligands, as protein was kept rigid and ligand was flexible. To better understand the stability and interactions of **A14** and lipase under the conditions of the surrounding environment, the 50 ns molecular dynamics simulation of ligand **A14**–protein complex was performed by using GROMACS software [53]. Simulation of protein without ligand **A14** (apo-protein) was also conducted under identical conditions, to compare with protein–ligand complex. The structural changes and dynamic behavior of protein–ligand complex were analyzed through RMSD (root mean square deviation), RMSF (root mean square fluctuation), Rg (radius of gyration), SASA (solvent accessibility surface area) values, and hydrogen-bond variations.

**Figure 10:**
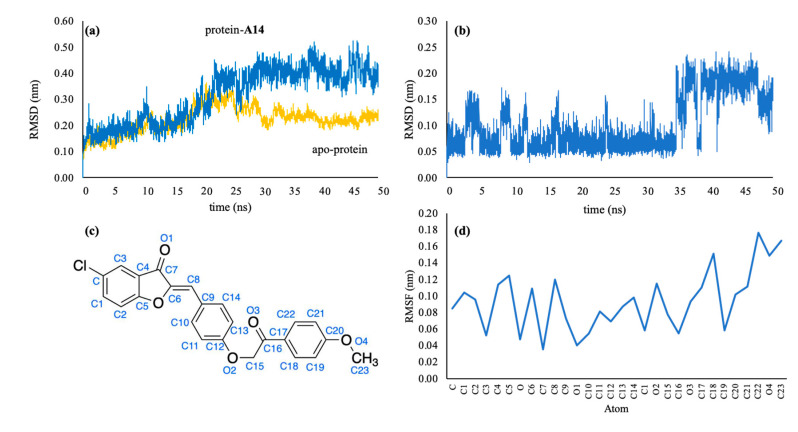
Stability of ligand **A14** in protein-**A14** during 50 ns molecular dynamics (MD) simulations. (**a**) Root mean square deviation (RMSD) values of 1LPB protein in apo-protein and protein–**A14** complex; (**b**) RMSD values of ligand A14 in protein–**A14** complex; (**c**) 2D structure of **A14** with atomic numbering; and (**d**) Root mean square fluctuation (RMSF) heavy chain atoms of ligand **A14**.

The stability of the 1LBP–**A14** complex was investigated by comparing RMSD and RMSF values of the protein–ligand complex with the corresponding values of the initial apo-protein. RMSD measures the average change of atoms displacement for a protein–ligand complex compared to the initial apo-protein structure. RMSD values were calculated for all frames in the MD simulation trajectory. The 1LBP–**A14** complex reached stability after 25 ns (RMSD values ranged from 2.5 to 4.0 Å (or 0.25 to 0.40 nm)) while the apo-protein was relatively stable after 30 ns. The RMSD value of ligand had an amplitude of oscillation about 1.5 Å (from 0.5 to 2.0 Å), showing that the structure of ligand **A14** was always stable during 50 ns simulation (Figure 10).

**Figure 11:**
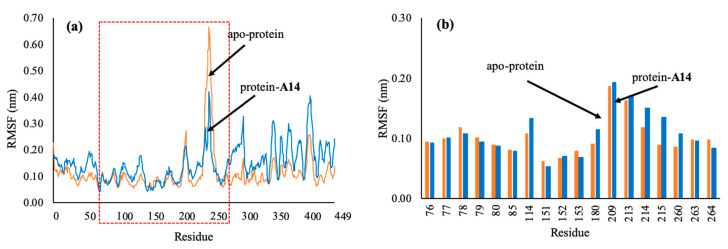
Root mean square fluctuation (RMSF) values of protein 1LPB during 50 ns MD simulations: (**a**) RMSF values of amino acids of protein 1LPB; (**b**) RMSF values of key residues in the active site of protein 1LPB. The protein-**A14** complex and the apo-protein 1LBP were showed in blue and orange, respectively.

Another stability factor is the fluctuations of protein during simulation that were evaluated by analyzing RMSF values (Figure 11). RMSF values characterize changes in both the protein chain and the ligand atoms. RMSF values of all atoms of the ligand **A14** were less than 2 Å. The oxygen atom of ligand **A14** (O2), which created hydrogen bonds with Ser152 and His263 has the RMSF value of 1.2 Å, while atom O3 has the RMSF value of 0.6 Å. The data demonstrated that atoms O2 and O3 created stable bonds with protein. The fluctuations of Ser152, His263, and Phe77 residues during the interaction were all below 2.0 Å, which are perfectly acceptable (Figure 10). On RMSF values, amino acids of the apo-protein were more fluctuated than those of the ligand **A14**–protein complex. Residues of lipase active site interacting with ligand **A14** were all stable with RMSF values below 2.0 Å; especially, Ser152, His263, and Phe77 had RMSF values below 1.0 Å. It could be inferred that the interactions of protein and ligand made the structure of protein gaining more stability than the apo-protein and that ligand **A14** fitted well in the active site and kept interacting with Ser152.

**Figure 12:**
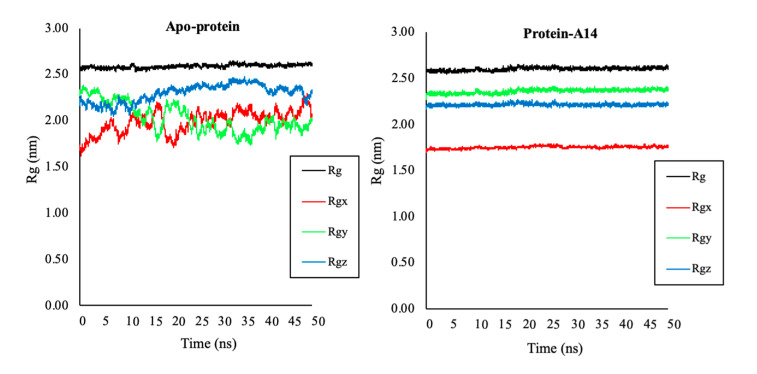
Radius of gyration (Rg) of protein 1LPB in apo-protein and protein–**A14** complex during 50 ns MD simulations: (**a**) Rg of protein in the protein-**A14** complex; (**b**) Rg of protein in the apo-protein. Total Rg of protein was in black, Rgx, Rgy, and Rgz around the *x*-, *y*-, and *z*-axis were in red, green, blue, respectively.

**Figure 13:**
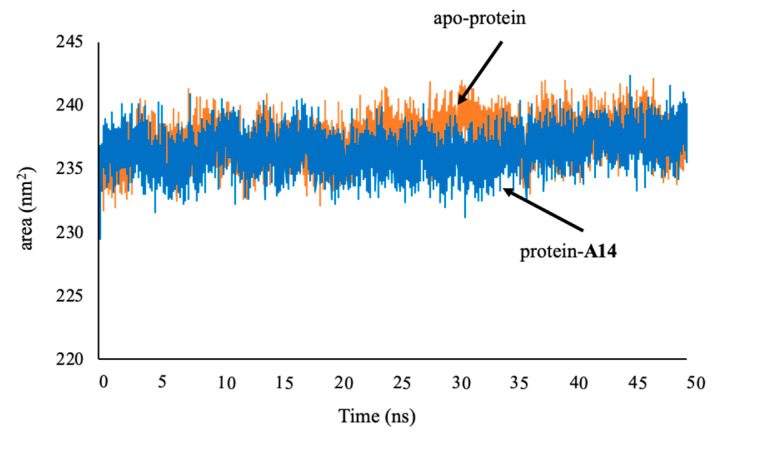
Total solvent-accessible surface area (SASA) of protein 1LPB in apo-protein and protein–**A14** complex.

Moreover, the rigidity of the protein system after the MD simulation was examined via the Rg values. The data showed that Rg average values of protein in the 1LPB–**A14** complex and of apo-protein were both about 2.6 nm, meaning that protein retained a stable structure during the MD simulations (Figure 12). SASA values evaluate the ability of bimolecular surface area assessable to solvent molecules (Figure 13). After 1 ns, SASA values of the protein in the complex and apo-protein reached the maximum values. The average values were 235 and 238 nm^2^, respectively. The lower SASA value of the protein in the ligand–protein complex denotes its relatively shrunken nature compared to the unbound structure.

Protein and ligand interactions can be further monitored throughout the simulations. These interactions included hydrogen bonds, hydrophobic interactions, ionic interactions, and water bridges. Hydrogen bonds play a crucial role in ligand binding. The occupancies of hydrogen bonds between ligand **A14** and protein 1LPB were determined by VMD software (d ≤ 3.5 Å and α ≤ 120°). Ligand **A14** plays the role of both donor and acceptor in forming hydrogen bonds with the protein. **A14** showed strong hydrogen bond interaction with the residue Phe77 (occupancy of 70.11%) and moderate hydrogen bond interaction with Ser152 (occupancy of 56.74%). Ligand **A14** also interacted with residues His263, Ala259, Arg256, Pro180, Leu153, Ser152, His151, Phe115, Tyr114, Ile78, and Phe77, although these hydrogen bonds were weak (occupancies less than 10%). The data proved that ligand **A14** bound well and relative stably in the catalytic cavity of the HPL during the 50 ns MD simulation.

On the molecular docking and MD results, ligand **A14** is a potential inhibitor as the ligand binding to the active site of protein 1LPB and mostly interacted with the key residues of the catalytic site (Ser152 and Phe77) by hydrogen bonds during the 50 ns MD simulation. The results of this study imply the potency of compound **A14** for inhibiting HPL.

## 3. Materials and Methods

### 3.1. Preparation of Protein and Ligand for Docking

Protein: The 3D structure of human pancreatic lipase was retrieved from the Protein Data Bank (PDB ID: 1LPB—resolution: 2.46 Å) (https://www.rcsb.org). The protein has a co-crystallized ligand—methoxy undecyl phosphonic acid. From the retrieved structure, protein chains binding with co-crystallized ligand was extracted, and then added polarized hydrogens by AutoDock Tools 1.5.7rc1 (Molecular Graphics Laboratory - The Scripps Research Institute, La Jolla, CA, USA).

Ligand: Ligands in this study were 82 aurone derivatives collected from reported publications investigating the biological effects of aurones [34,35,36,37,38,39,40,41,42,43,44,45,46,47,48,49]. These compounds were drawn in 2D by ISIS Draw 2.5 program and formatted in the MOL file. All 2D structures were converted to 3D structures whose energies were minimized with the YASARA Energy Minimization server (https://www.yasara.org—YASARA Biosciences GmbH, Vienna, Austria).

### 3.2. Molecular Docking

The molecular docking process was implemented by AutoDock Vina software version 1.1.2, (Molecular Graphics Laboratory, The Scripps Research Institute, La Jolla, CA, USA) which combines knowledge-based potentials and empirical scoring [50]. The binding site parameters (x: 8,431 Å; y: 24,417 Å; z: 52,623 Å) and the docking box dimensions (18 × 18 × 18 Å) were determined by redocking the co-crystallization ligand in the catalytic cavity. The results of molecular docking were evaluated through the criteria of binding structure, binding energy, and possible interactions between ligand and the key residues of the protein.

### 3.3. Molecular Dynamics Simulations

GROMACS 2018.01 software (Department of Biophysical Chemistry, University of Groningen, Groningen, The Netherlands) was used for the MD simulations of protein without ligand (apo-protein) and protein in complex with a ligand [53]. From the result of docking, choosing the best configuration of the best binding compound, and then adding hydrogen by Avogadro ver. 1.2.0n software (Avogadro development team, New York, NY, USA). The topology of the ligand structure was created by CGENFF with force field CHARMM27. The test system was put in a simulation box, and it was placed 1.0 nm from the box edge, which was a 12-surface polyhedron and contained the water solvent of the TIP3P model. Na^+^, Cl^−^ ions were added to the system to balance the charge. The process of energy minimization took place to eliminate the negative interactions in the system. The system was balanced under the “isothermal-isobaric” conditions NVT (N = number of particles, V = volume, and T = temperature 300 K) and NPT (N = number of particles, P = pressure 1 bar, and T = temperature 300 K). Berendsen thermostat and Parrinello–Rahman barostat were used to maintain temperature and pressure. This equilibrium process lasted 1000 ps for each NVT and NPT system. The systems were simulated for 50 ns, at a temperature of 300 K, and pressure of 1 bar.

The result was recorded every 0.01 ns and evaluated through RMSD (root mean square deviation), RMSF, Rg, and SASA. Parameters were calculated by software GROMACS, VMD version 1.9.3 (Theoretical and Computational Biophysics Group, Beckman Institute for Advanced Science and Technology, University of Illinois at Urbana, IL, USA) [54,55], and represented as a chart by Microsoft Excel 2016 (Microsoft—COMDEX, Las Vegas, NV, USA).

## 4. Conclusions

Among 82 bioactive aurone derivatives, compound **A14** ((*Z*)-5-chloro-2-(4-(2-(4-methoxyphenyl)-2-oxoethoxy) benzylidene) benzofuran-3(2*H*)-one) stands out as a potential candidate for inhibiting human pancreatic lipase. **A14** was docked into the HPL binding site with the highest docking score (−10.6 kcal⋅mol^−1^). In 50 ns molecular dynamics simulations, **A14** fitted well in the catalytic cavity of protein 1LPB and maintained the interactions by hydrogen bonds and hydrophobic interactions with the key residues of the catalytic site. Further studies on the synthesis of **A14** and its derivatives, as well as in vitro lipase inhibitory assay are under investigation.

## Figures and Tables

**Table 1:** Docking profiles of methoxy undecyl phosphonic acid (MUP), orlistat, and the general structure of aurones.

Compound	DS ^1^ (kcal⋅mol^−1^)	Hydrogen Bonds	Hydrophobic Interactions
MUP	−4.3	Ser152, Phe77, Leu153, His263	Tyr114, Pro180, Phe215
Orlistat	−6.7	Ser152, Phe77, Asp79, His151	Phe77, Tyr114, His263
**A0**	−8.8	Ser152, His263	Phe77, Tyr114, Pro180, Phe215

^1^ Docking score.

**Table 2:** Docking scores of aurone derivatives in Group I and Group II.

Compound	Docking Scores (kcal⋅mol^−1^)	Compound	Docking Scores (kcal⋅mol^−1^)	Compound	Docking Scores (kcal⋅mol^−1^)
*Group I*				*Group II*	
**A1–A8**	−8.8 to −10.1	**A30–A36**	−8.4 to −9.1	**A63–A64**	−9.2 to −9.4
**A9–A13**	−8.6 to −10.5	**A37–A44**	−8.3 to −10.5	**A65–A77**	−7.4 to −10.1
**A14–A16**	−9.9 to −10.6	**A45–A56**	−8.2 to −9.7	**A78–A82**	−8.3 to −8.8
**A17–A29**	−8.3 to −10.2	**A57–A62**	−8.2 to −10.1		

## Supplementary Materials

The following are available online. Table S1. Docking results of 82 aurone compounds with protein.
