# Supplementary material for: Exploring Aurone Derivatives as Potential Human Pancreatic Lipase Inhibitors through Molecular Docking and Molecular Dynamics Simulations
Source: Molecules. 2020 Oct 13;25(20):4657. doi: 10.3390/molecules25204657 (PMC7587340; doi:10.3390/molecules25204657)
Supplement: Supplementary file 1 [file molecules-25-04657-s001.pdf]

Table S1. Docking results of 82 aurone compounds with protein (PDB ID: 1LPB).

	Structure	IUPAC name	Docking score kcal.mol^{-1}	Hydrogen bonds	Hydrophobic interaction
**A1**	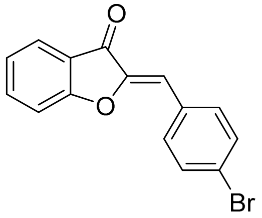	(Z)-2-(4-bromobenzylidene)benzofuran-3(2H)-one	-9.1	Ser152<br>His263	Phe77<br>Phe215
**A2**	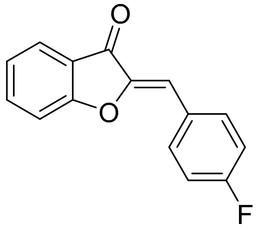	(Z)-2-(4-fluorobenzylidene)benzofuran-3(2H)-one	-9.1	Phe77<br>His263	Phe77<br>His263
**A3**	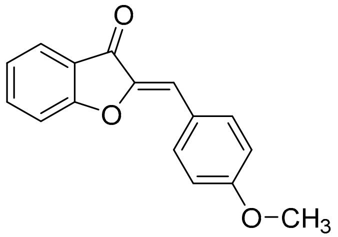	(Z)-2-(4-methoxybenzylidene)benzofuran-3(2H)-one	-9.3	Phe77	His263<br>Tyr114
**A4**	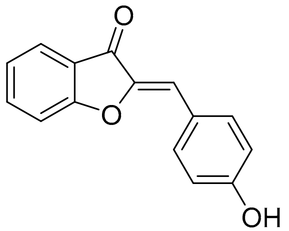	(Z)-2-(4-hydroxybenzylidene)benzofuran-3(2H)-one	-8.9	Ser152	Phe215<br>Pro180

**A5**	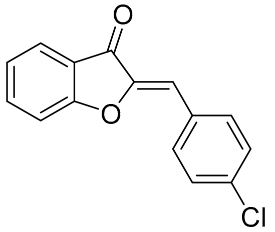	(Z)-2-(4-chlorobenzylidene)benzofuran-3(2H)-one	-9.0	Ser152<br>His263	Phe77<br>Arg256<br>Phe215
**A6**	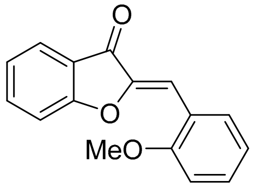	(Z)-2-(2-methoxybenzylidene)benzofuran-3(2H)-one	-9.0	Phe77	His263<br>Tyr114
**A7**	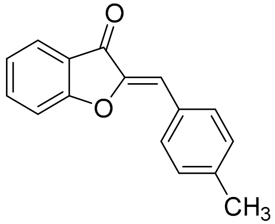	(Z)-2-(4-methylbenzylidene)benzofuran-3(2H)-one	-10.1	Phe77	His263<br>Tyr114
**A8**	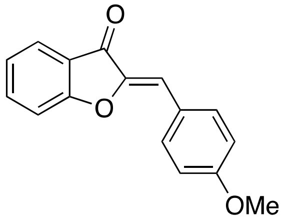	(*Z*)-2-(4-methoxybenzylidene)benzofuran-3(2*H*)-one	-9.4	Phe77<br>Arg256	His263<br>Phe215
**A9**	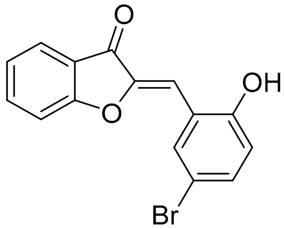	(Z)-2-(5-bromo-2-hydroxybenzylidene)benzofuran-3(2H)-one	-10.2	Ser152<br>His263<br>His151<br>Phe215	Phe215<br>Tyr114

**A10**	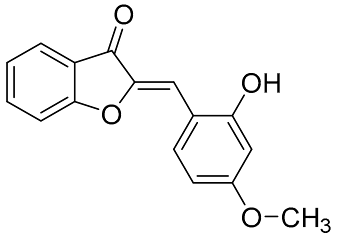	(Z)-2-(2-hydroxy-4-methoxybenzylidene)benzofuran-3(2H)-one	-9.5	Ser152<br>Phe77	Tyr114<br>Phe215
**A11**	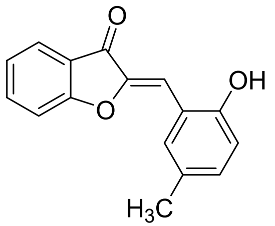	(Z)-2-(2-hydroxy-5-methylbenzylidene)benzofuran-3(2H)-one	-10.5	Ser152<br>His263<br>His151<br>Phe215	Phe77
**A12**	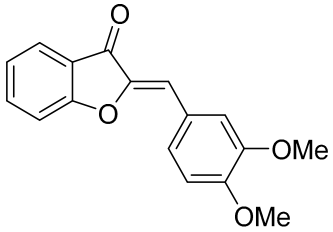	(Z)-2-(3,4-dimethoxybenzylidene)benzofuran-3(2H)-one	-8.6	Ser152<br>His263	Phe77<br>Ile78
**A13**	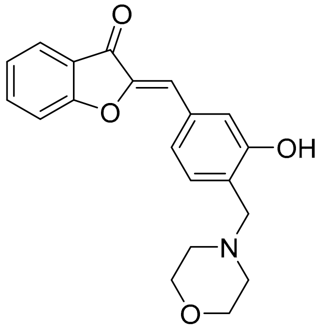	(Z)-2-(3-hydroxy-4-(morpholinomethyl)benzylidene)benzofuran-3(2H)-one	-8.8	Ser152<br>His263	Phe77

**A14**	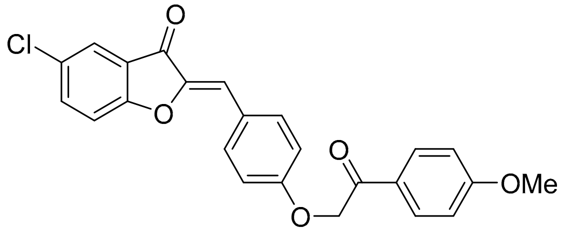	(Z)-5-chloro-2-(4-(2-(4-methoxyphenyl)-2-oxoethoxy)benzylidene)benzofuran-3(2H)-one	-10.6	Ser152<br>His263<br>His151	Phe215<br>Leu264
**A15**	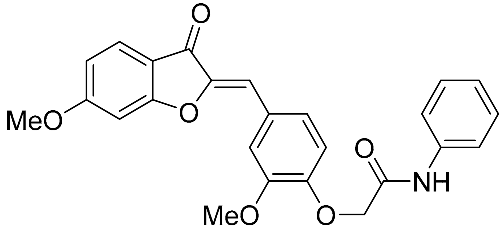	(Z)-2-(2-methoxy-4-((6-methoxy-3-oxobenzofuran-2(3H)-ylidene)methyl)phenoxy)-N-phenylacetamide	-9.8	His263	Phe77<br>Phe215
**A16**	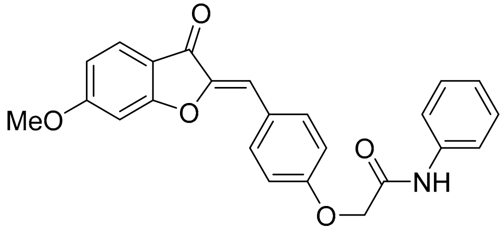	(Z)-2-(4-((6-methoxy-3-oxobenzofuran-2(3H)-ylidene)methyl)phenoxy)-N-phenylacetamide	-9.9	Ser152<br>His151<br>His263	Phe215<br>Tyr114<br>Arg256
**A17**	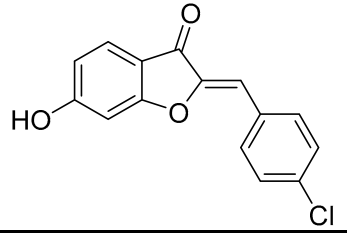	(Z)-2-(4-chlorobenzylidene)-6-hydroxybenzofuran-3(2H)-one	-9.3	Ser152<br>His263	Tyr114<br>Pro180

**A18**	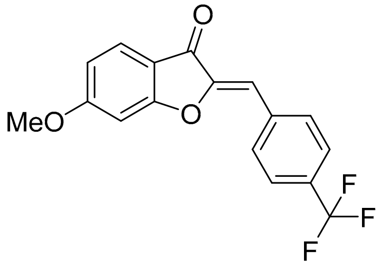	(Z)-6-methoxy-2-(4-(trifluoromethyl)benzylidene)benzofuran-3(2H)-one	-10.2	Ser152	His263<br>Phe77<br>Pro180
**A19**	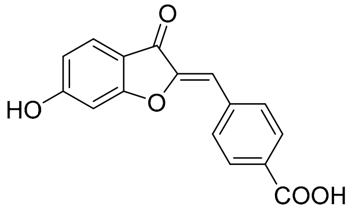	(Z)-4-((6-hydroxy-3-oxobenzofuran-2(3H)-ylidene)methyl)benzoic acid	-8.8	Ser152<br>His263	Phe77
**A20**	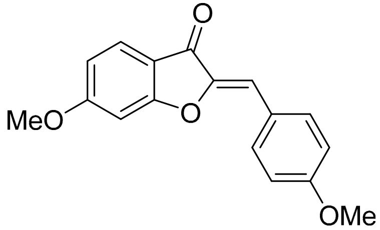	(Z)-6-methoxy-2-(4-methoxybenzylidene)benzofuran-3(2H)-one	-9.2	Phe77<br>Agr256	His263<br>Phe215
**A21**	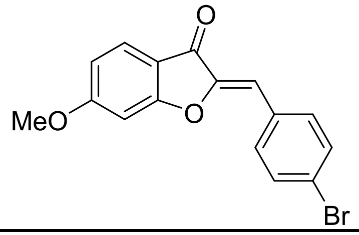	(Z)-2-(4-bromobenzylidene)-6-methoxybenzofuran-3(2H)-one	-8.9	Ser152<br>His263	Phe77<br>Tyr114

**A22**	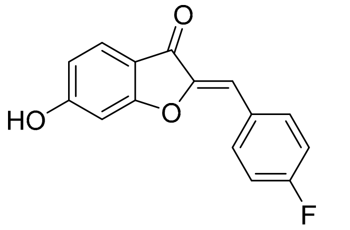	(Z)-2-(4-fluorobenzylidene)-6-hydroxybenzofuran-3(2H)-one	-9.7	Phe77	His263<br>Tyr114<br>Pro180
**A23**	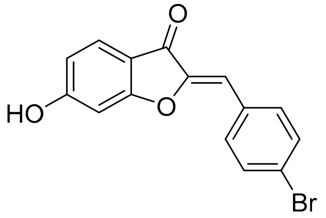	(Z)-2-(4-bromobenzylidene)-6-hydroxybenzofuran-3(2H)-one	-9.5	Phe77	His263<br>Tyr114<br>Pro180
**A24**	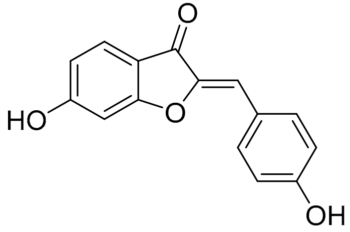	(Z)-6-hydroxy-2-(4-hydroxybenzylidene)benzofuran-3(2H)-one	-8.3	Ser152<br>His263	Phe77
**A25**	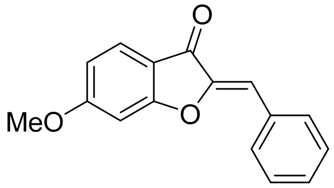	(Z)-2-benzylidene-6-methoxybenzofuran-3(2H)-one	-9.7	Phe77	His263<br>Tyr114<br>Pro180
**A26**	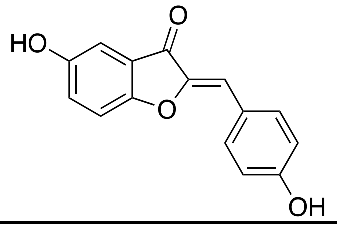	(*Z*)-5-hydroxy-2-(4-hydroxybenzylidene)benzofuran-3(2*H*)-one	-9.9	Ser152<br>His151<br>His263<br>Asp79	Tyr114

**A27**	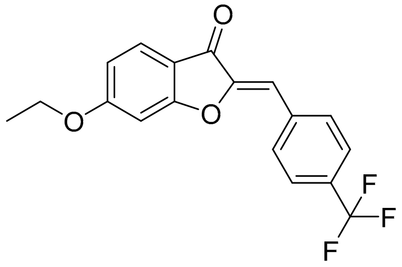	(Z)-6-ethoxy-2-(4-(trifluoromethyl)benzylidene)benzofuran-3(2H)-one	-10.1	Ser152<br>Phe77	His263<br>Pro180
**A28**	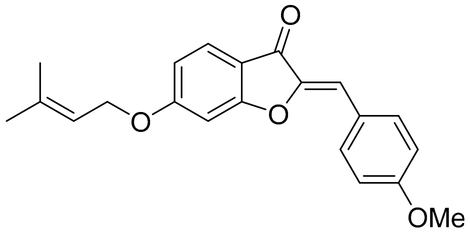	(Z)-2-(4-methoxybenzylidene)-6-((3-methylbut-2-en-1-yl)oxy)benzofuran-3(2H)-one	-10.0	Phe77	His263<br>Ile78<br>Tyr114
**A29**	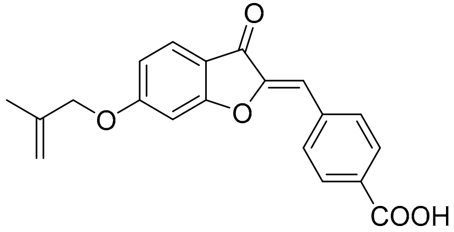	(Z)-4-((6-((2-methylallyl)oxy)-3-oxobenzofuran-2(3H)-ylidene)methyl)benzoic acid	-9.9	Ser152<br>His263<br>Arg256	Phe77<br>Phe215
**A30**	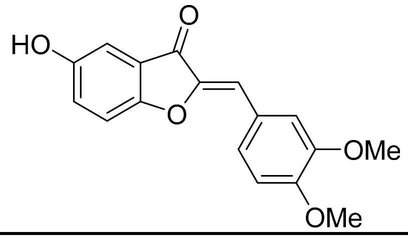	(Z)-2-(3,4-dimethoxybenzylidene)-5-hydroxybenzofuran-3(2H)-one	-8.5	Phe77<br>His263	His263<br>Phe215

**A31**	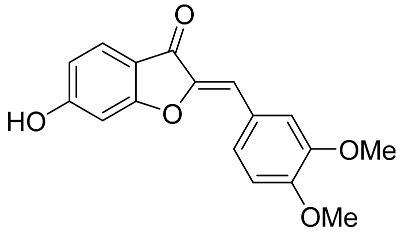	(Z)-2-(3,4-dimethoxybenzylidene)-6-hydroxybenzofuran-3(2H)-one	-8.4	Ser152<br>His263	Phe77<br>Phe215
**A32**	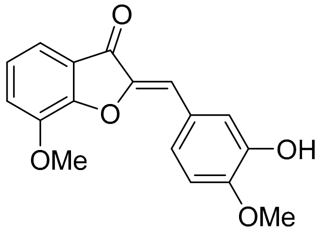	(Z)-2-(3-hydroxy-4-methoxybenzylidene)-7-methoxybenzofuran-3(2H)-one	-9.0	Phe77	His263<br>Tyr114
**A33**	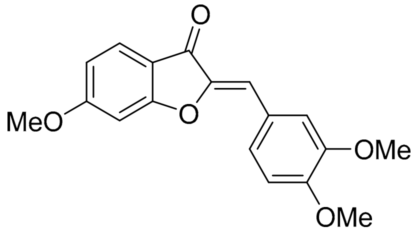	(Z)-2-(3,4-dimethoxybenzylidene)-6-methoxybenzofuran-3(2H)-one	-8.5	Ser152<br>Arg256	His263<br>Phe77
**A34**	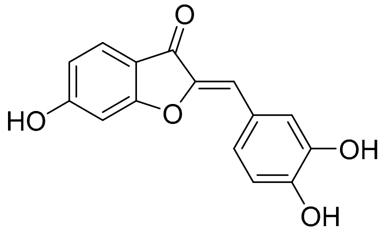	(Z)-2-(3,4-dihydroxybenzylidene)-6-hydroxybenzofuran-3(2H)-one	-8.9	Phe77	His263<br>Tyr114
**A35**	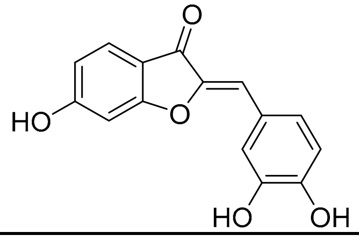	(Z)-2-(3,4-dihydroxybenzylidene)-6-hydroxybenzofuran-3(2H)-one	-8.9	Phe77	His263<br>Tyr114

**A36**	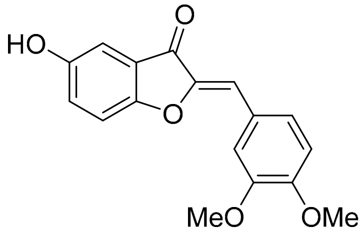	(Z)-2-(3,4-dimethoxybenzylidene)-5-hydroxybenzofuran-3(2H)-one	-9.1	Phe77	His263<br>Phe215
**A37**	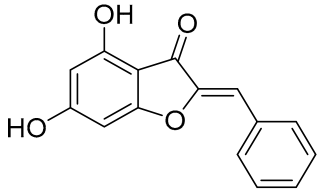	(Z)-2-benzylidene-4,6-dihydroxybenzofuran-3(2H)-one	-9.9	Phe77<br>Asp79	His263<br>Tyr114
**A38**	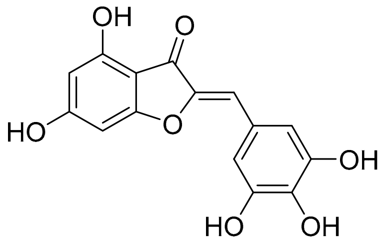	(Z)-4,6-dihydroxy-2-(3,4,5-trihydroxybenzylidene)benzofuran-3(2H)-one	-9.7	Ser152<br>Agr256	phe77
**A39**	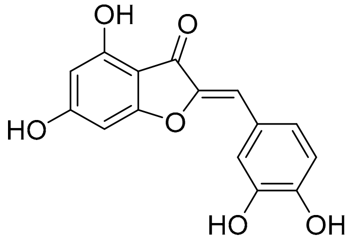	(Z)-2-(3,4-dihydroxybenzylidene)-4,6-dihydroxybenzofuran-3(2H)-one	-9.2	Phe77<br>His151	His263
**A40**	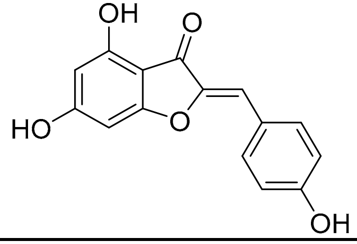	(Z)-4,6-dihydroxy-2-(4-hydroxybenzylidene)benzofuran-3(2H)-one	-9.4	Ser152<br>Phe77	His263<br>Tyr114

**A41**	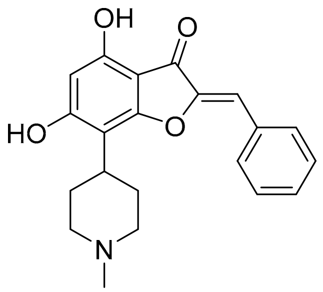	(Z)-2-benzylidene-4,6-dihydroxy-7-(1-methylpiperidin-4-yl)benzofuran-3(2H)-one	-9.8	Ser152<br>His263	Phe77
**A42**	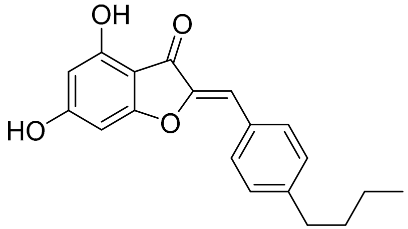	(Z)-2-(4-butylbenzylidene)-4,6-dihydroxybenzofuran-3(2H)-one	-10.5	Ser152<br>His263	Phe215<br>Tyr114
**A43**	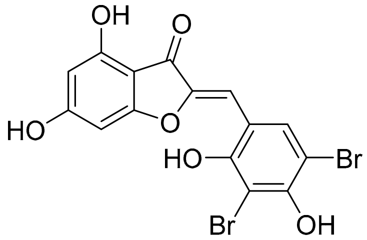	(Z)-2-(3,5-dibromo-2,4-dihydroxybenzylidene)-4,6-dihydroxybenzofuran-3(2H)-one	-8.2	His263	Phe77<br>Phe215<br>Ile209
**A44**	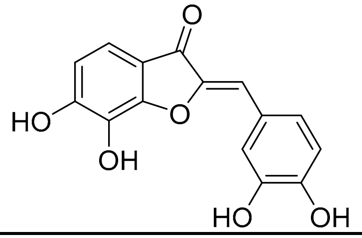	(Z)-2-(3,4-dihydroxybenzylidene)-6,7-dihydroxybenzofuran-3(2H)-one	-8.8	Phe77	His263<br>Tyr114

**A45**	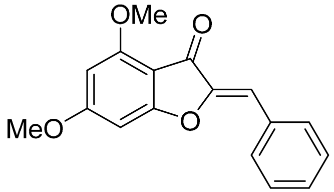	(Z)-2-benzylidene-4,6-dimethoxybenzofuran-3(2H)-one	-8.7	Ser152<br>His263	Phe77
**A46**	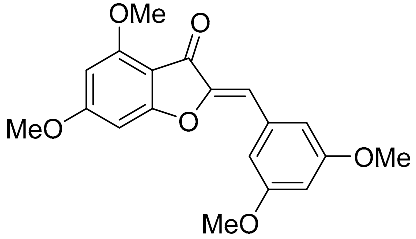	(Z)-2-(3,5-dimethoxybenzylidene)-4,6-dimethoxybenzofuran-3(2H)-one	-8.4	Ser152<br>Phe77<br>His263	Phe77<br>Tyr114
**A47**	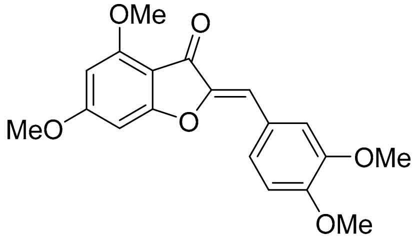	(Z)-2-(3,4-dimethoxybenzylidene)-4,6-dimethoxybenzofuran-3(2H)-one	-8.2	Ser152<br>Phe77<br>His263	Phe77<br>Tyr114
**A48**	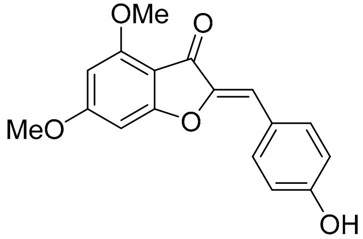	(Z)-2-(4-hydroxybenzylidene)-4,6-dimethoxybenzofuran-3(2H)-one	-9.7	Ser152<br>Phe77<br>His263<br>Asp79<br>Arg256	His263
**A49**	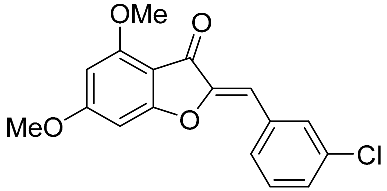	(Z)-2-(3-chlorobenzylidene)-4,6-dimethoxybenzofuran-3(2H)-one	-8.9	Asp79	His263<br>Tyr114

**A50**	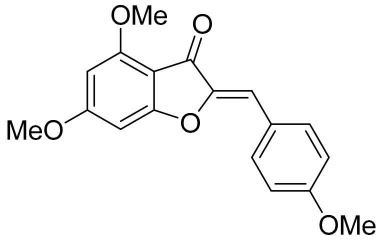	(Z)-4,6-dimethoxy-2-(4-methoxybenzylidene)benzofura n-3(2H)-one	-8.7	Ser152<br>Phe77	Tyr114<br>Phe215
**A51**	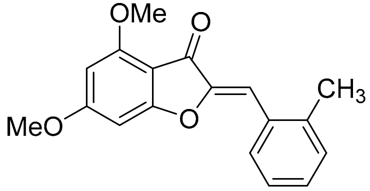	(Z)-4,6-dimethoxy-2-(2-methylbenzylidene)benzofuran-3(2H)-one	-8.7	Ser152<br>His263	Phe77<br>Ile78
**A52**	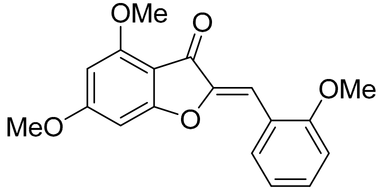	(Z)-4,6-dimethoxy-2-(2-methoxybenzylidene)benzofura n-3(2H)-one	-8.6	Phe77<br>Asp79	His263
**A53**	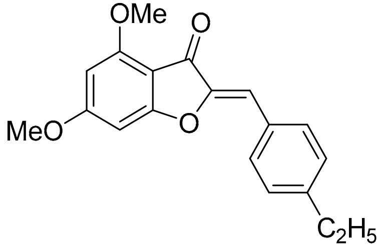	(Z)-2-(4-ethylbenzylidene)-4,6-dimethoxybenzofuran-3(2H)-one	-9.0	Ser152<br>His263<br>Phe215	Phe77
**A54**	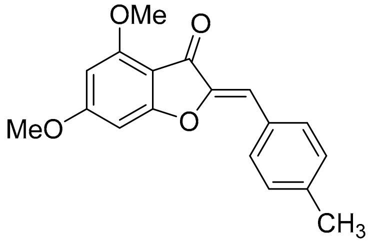	(Z)-4,6-dimethoxy-2-(4-methylbenzylidene)benzofuran-3(2H)-one	-9.6	Phe77<br>Asp79<br>Arg256	His263<br>Pro180

**A55**	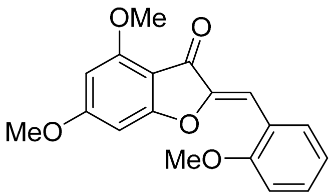	(Z)-4,6-dimethoxy-2-(2-methoxybenzylidene)benzofuran-3(2H)-one	-8.4	Phe77<br>Asp79<br>Agr256	His263
**A56**	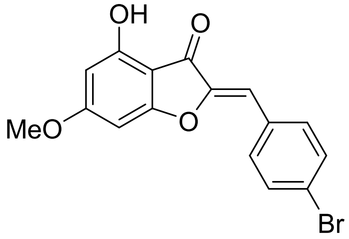	(Z)-2-(4-bromobenzylidene)-4-hydroxy-6-methoxybenzofuran-3(2H)-one	-9.1	His151<br>Asp79	Phe77
**A57**	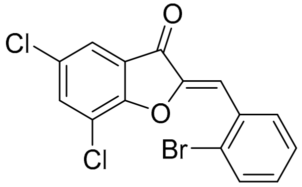	(Z)-2-(2-bromobenzylidene)-5,7-dichlorobenzofuran-3(2H)-one	-8.2	His263	Phe215<br>Phe77
**A58**	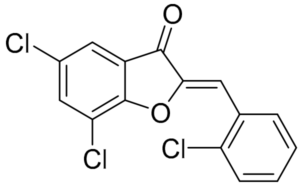	(Z)-5,7-dichloro-2-(2-chlorobenzylidene)benzofuran-3(2H)-one	-9.0	Ser152<br>His263	Tyr114<br>Phe215<br>Pro180
**A59**	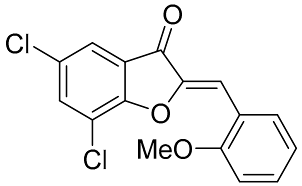	(Z)-5,7-dichloro-2-(2-methoxybenzylidene)benzofuran-3(2H)-one	-8.8	Phe 77	His 263<br>Arg 256

**A60**	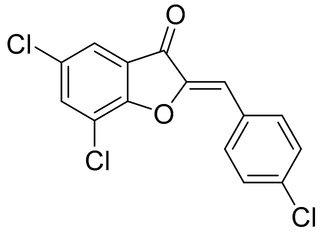	(Z)-5,7-dichloro-2-(4-chlorobenzylidene)benzofuran-3(2H)-one	-10.1	Ser152<br>His263	His263<br>Tyr114
**A61**	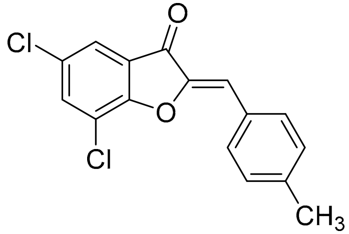	(Z)-5,7-dichloro-2-(4-methylbenzylidene)benzofuran-3(2H)-one	-10.0	Ser152<br>His263	Tyr114<br>Pro180
**A62**	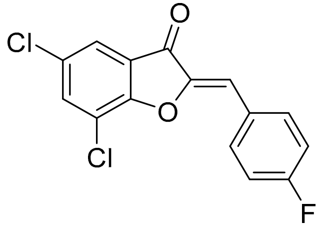	(Z)-5,7-dichloro-2-(4-fluorobenzylidene)benzofuran-3(2H)-one	-9.6	Ser152<br>Asp79<br>Arg256	His263<br>Phe77
**A63**	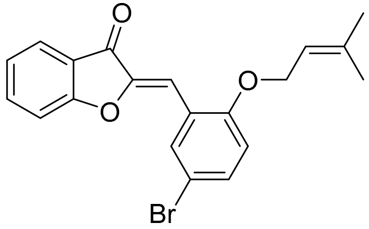	(Z)-2-(5-bromo-2-((3-methylbut-2-en-1-yl)oxy)benzylidene)benzofuran-3(2H)-one	-9.4		Phe77<br>Tyr114
**A64**	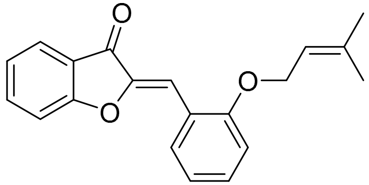	(Z)-2-(2-((3-methylbut-2-en-1-yl)oxy)benzylidene)benzofuran-3(2H)-one	-9.2		Leu213<br>Phe215<br>Pro180

**A65**	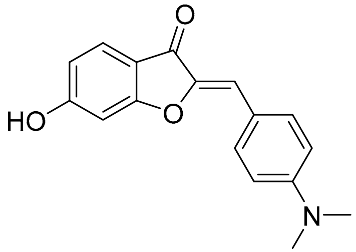	(Z)-2-(4-(dimethylamino)benzylidene)-6-hydroxybenzofuran-3(2H)-one	-9.0		Phe215<br>Pro180
**A66**	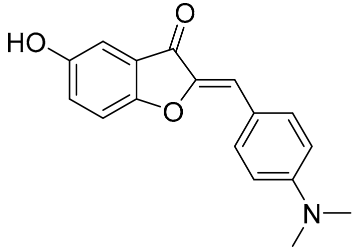	(Z)-2-(4-(dimethylamino)benzylidene)-5-hydroxybenzofuran-3(2H)-one	-10.1		His263<br>Phe215
**A67**	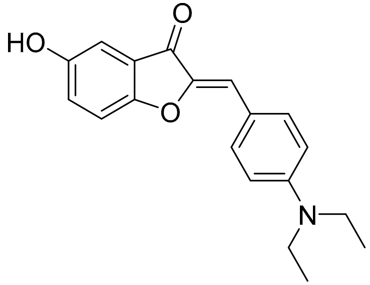 8	(Z)-2-(4-(diethylamino)benzylidene)-5-hydroxybenzofuran-3(2H)-one	-8.8	Asp79	Phe77<br>Tyr114
**A68**	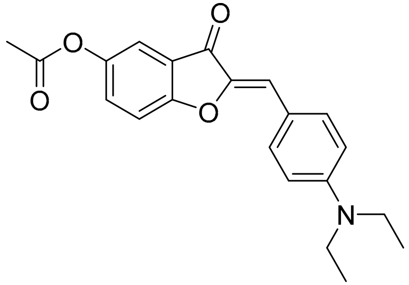	(Z)-2-(4-(diethylamino)benzylidene)-3-oxo-2,3-dihydrobenzofuran-5-yl acetate	-9.0	Asp79	Phe77<br>Phe215

**A69**	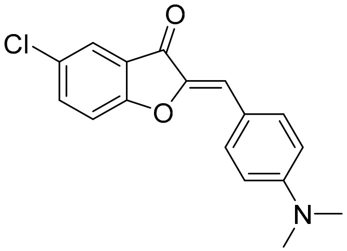	(Z)-5-chloro-2-(4-(dimethylamino)benzylidene)benzofuran-3(2H)-one	-9.0	Asp79	Tyr114<br>Phe215<br>Pro180
**A70**	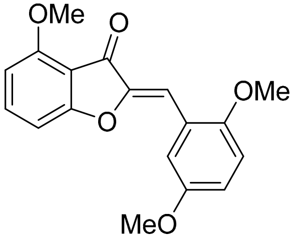	(Z)-2-(2,5-dimethoxybenzylidene)-4-methoxybenzofuran-3(2H)-one	-8.4		His263<br>Phe77<br>Phe215
**A71**	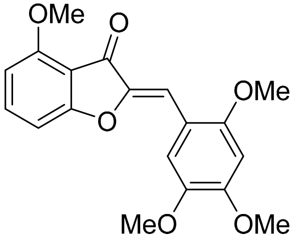	(Z)-4-methoxy-2-(2,4,5-trimethoxybenzylidene)benzofuran-3(2H)-one	-8.1	Phe215	Phe77<br>His263<br>Tyr114
**A72**	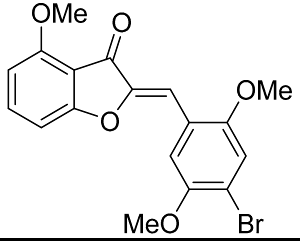	(Z)-2-(4-bromo-2,5-dimethoxybenzylidene)-4-methoxybenzofuran-3(2H)-one	-7.4		Phe215<br>Tyr 114

**A73**	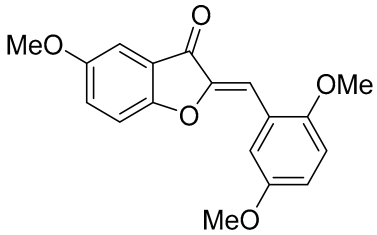	(Z)-2-(2,5-dimethoxybenzylidene)-5-methoxybenzofuran-3(2H)-one	-8.8	His151<br>Phe215	His263<br>Pro180
**A74**	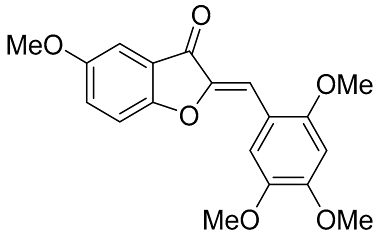	(Z)-5-methoxy-2-(2,4,5-trimethoxybenzylidene)benzofuran-3(2H)-one	-8.0	His263<br>His151<br>Phe215	Phe77<br>Arg256
**A75**	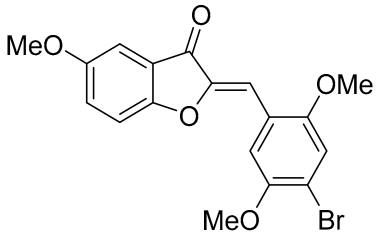	(Z)-2-(4-bromo-2,5-dimethoxybenzylidene)-5-methoxybenzofuran-3(2H)-one	-8.8		Tyr114<br>Pro180<br>Phe215
**A76**	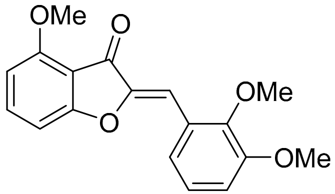	(Z)-2-(2,3-dimethoxybenzylidene)-4-methoxybenzofuran-3(2H)-one	-7.9		Phe 77<br>Arg256<br>Leu264
**A77**	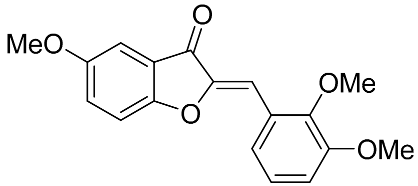	(Z)-2-(2,3-dimethoxybenzylidene)-5-methoxybenzofuran-3(2H)-one	-7.5		Pro180<br>Tyr114

**A78**	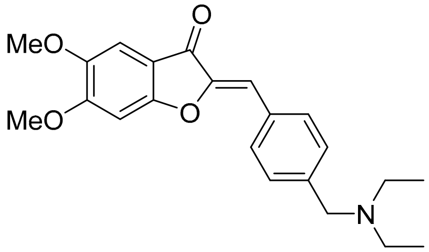	(Z)-2-(4-((diethylamino)methyl)benzylidene)-5,6-dimethoxybenzofuran-3(2H)-one	-8.6	Asp79	His263<br>Asp79<br>Phe215
**A79**	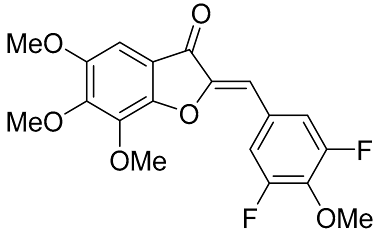	(Z)-2-(3,5-difluoro-4-methoxybenzylidene)-5,6,7-trimethoxybenzofuran-3(2H)-one	-8.8		Phe77<br>Phe215
**A80**	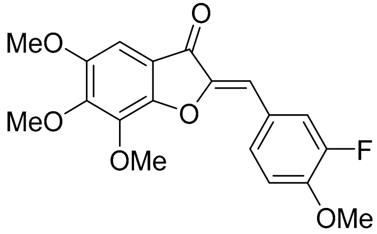	(Z)-2-(3-fluoro-4-methoxybenzylidene)-5,6,7-trimethoxybenzofuran-3(2H)-one	-8.5		Phe77<br>His263
**A81**	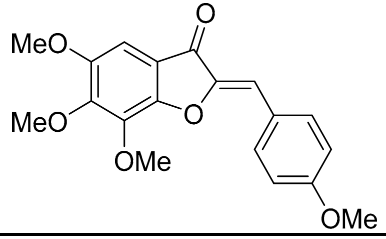	(Z)-5,6,7-trimethoxy-2-(4-methoxybenzylidene)benzofuran-3(2H)-one	-8.3		His263<br>Phe77

**A82**	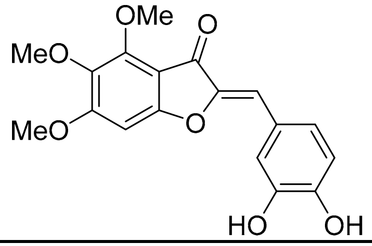	(Z)-2-(3,4-dihydroxybenzylidene)-4,5,6-trimethoxybenzofuran-3(2H)-one	-8,7	His263<br>Phe77	Phe77<br>Phe215
